# Disruption of Undergraduate Clinical Training in Postconflict Syria: Insights from Clinical Practice and Implications for Future Health Workforce Readiness

**DOI:** 10.1055/s-0046-1827807

**Published:** 2026-08-29

**Authors:** Rawan Al-Deeb, Taim Al Kheder, Mohammad Al-Akchar

**Affiliations:** 1 Faculty of Medicine, Homs University, Homs, Syrian Arab Republic; 2 Faculty of Medicine, Damascus University, Damascus, Syrian Arab Republic; 3 Division of Cardiology, Department of Internal Medicine, Southern Illinois University School of Medicine, Springfield, Illinois, United States

**Keywords:** undergraduate medical education, clinical training, medical students, future health workforce, Syria

## Abstract

Clinical training is a fundamental component of medical education and plays a critical role in preparing future physicians for clinical practice. In Syria, the prolonged effects of conflict continue to shape health care institutions and training environments despite the decline of large-scale hostilities. Drawing on firsthand observations from clinical-stage medical students at Syrian public universities, we highlight recent changes in undergraduate clinical training, together with recent policy changes that discontinued practical assessments for fourth- and fifth-year students and concentrated formal clinical assessment within the final year of study. While these changes may reflect resource and institutional constraints, they may also limit opportunities for the gradual development and reinforcement of clinical competencies. Given existing evidence of gaps in practical preparedness among Syrian medical students, preserving meaningful clinical exposure and competency-based assessment throughout the clinical years should remain a priority for medical schools and policymakers as Syria rebuilds its future health workforce.

## Why Clinical Training Matters

Undergraduate clinical training is a cornerstone of medical education, enabling students to integrate theoretical knowledge with clinical reasoning, practical skills, and professional development through supervised participation in patient care. Effective clinical training depends on structured supervision, continuity of clinical exposure, and progressive participation in patient care, all of which support competency development and readiness for independent practice. For undergraduate medical students, workplace learning spans a continuum of clinical experiences, from early patient encounters to clerkships, serving as a key mechanism through which knowledge, skills, and professional behaviors are developed. 1 2 3 4

## Clinical Training in Postconflict Syria

Over the past decade, the Syrian conflict has profoundly affected health care delivery, medical infrastructure, and medical education through the destruction of facilities, displacement of health care professionals, and disruption of academic institutions. 5 6 Although large-scale hostilities have declined in many regions, medical schools and teaching hospitals continue to operate within a postconflict environment characterized by resource limitations, workforce shortages, and uneven institutional capacity. 5 6 7 Indeed, these challenges are evident even in major academic centers, for example, Al-Mouwasat University Hospital, one of Syria's leading teaching and referral hospitals, continues to face shortages in essential medications, diagnostic services, equipment, and clinical resources that affect both patient care and training environments. 6 They extend beyond educational infrastructure alone and continue to shape the quality, consistency, and accessibility of undergraduate clinical training experiences.

## Insights from Clinical Practice

As current clinical-stage medical students at Syrian public universities, we have observed substantial changes in the structure of undergraduate clinical training during the 2025–2026 academic year. Medical education in Syria consists of a 6-year program, with the final 3 years traditionally representing the clinical phase. Historically, clinical clerkships were conducted throughout the fourth, fifth, and sixth years, providing students with progressive exposure to patient care and clinical environments and allowing practical competencies to be assessed through formal clinical interviews and objective structured clinical examinations (OSCEs).

In November 2025, the Syrian Arab Republic Council of Higher Education (Decision No. 119) introduced changes to the assessment system for faculties of medicine at public universities. The decision discontinued OSCEs and interview-based clinical assessments for fourth- and fifth-year medical students and made attendance at hospital-based sessions a prerequisite for sitting the end-of-semester theoretical examinations. The decision applies to all public faculties of medicine in Syria (Damascus, Aleppo, Latakia, Homs, Hama, Tartous, Idlib, and Al-Furat Universities).

From our firsthand experience as current clinical-stage medical students, undergraduate clinical teaching during the 2025–2026 academic year has increasingly shifted away from traditional bedside clerkships toward predominantly classroom-based sessions conducted within teaching hospitals. Rather than participating in regular ward rounds, patient history-taking, physical examination, and supervised bedside discussions, students often attend didactic sessions resembling theoretical lectures, with comparatively limited opportunities for direct patient interaction or hands-on clinical skills development. Under Decision No. 119, attendance at hospital-based sessions is required for eligibility to sit the end-of-semester theoretical examinations, whereas clinical competencies are no longer formally assessed through OSCEs or clinical interviews during the fourth and fifth years.

By contrast, sixth-year students continue to undertake extensive clinical rotations across multiple specialties, often revisiting disciplines that were taught theoretically during the fourth and fifth years. While this model may preserve clinical exposure before graduation, it compresses workplace-based learning into a shorter period and may reduce opportunities for the gradual development and reinforcement of clinical competencies.

## Implications for Future Health Workforce Readiness

Clinical competence develops through repeated patient encounters, supervised practice, feedback, and progressive responsibility. 1 2 3 4 Recent evidence from Syria has identified important gaps in practical preparedness among medical students, including limited first-aid knowledge, insufficient basic life support awareness, and perceived deficiencies in training quality that have prompted calls for improved clinical training. 7 8 9

These findings matter because reducing longitudinal clinical engagement during the fourth and fifth years may further limit the gradual integration of theoretical knowledge with practical skills such as history-taking, physical examination, emergency response, clinical reasoning, and patient communication.

Against the backdrop of these recent changes in undergraduate clinical training, some educators and university administrators have argued that, because most contemporary medical graduates now pursue residency training, extensive undergraduate clinical exposure is less essential than in previous generations. However, residency training should build upon, rather than replace, competencies acquired during undergraduate medical education. Foundational clinical skills (including history-taking, physical examination, communication, basic procedural skills, and first-aid competencies) are developed through repeated practice and reinforcement over time, not through isolated or compressed periods of clinical exposure. While concentrating clinical training within the final year is a model some systems adopt deliberately and may offer advantages, such as greater continuity within individual clerkships and reduced fragmentation of clinical placements, it also compresses workplace-based learning into a shorter period and places substantial pressure on both students and clinical educators. This may reduce opportunities for the gradual development, reinforcement, and integration of clinical competencies that are typically acquired through repeated patient encounters over several years.

## A Call for Action

As Syria continues to rebuild its health care system in the postconflict era, strengthening undergraduate clinical training should be recognized as a workforce development priority. Medical schools and policymakers should strive to preserve meaningful clinical exposure and practical assessment throughout the clinical years, even within resource-constrained settings. In particular, the implications of Decision No. 119 issued by the Syrian Arab Republic Council of Higher Education should be carefully reconsidered in light of its potential impact on competency development and future health workforce preparedness. Particular attention should be directed toward maintaining opportunities for supervised patient encounters, skills-based learning, and competency-focused evaluation. Ensuring that future physicians graduate with adequate clinical confidence and practical preparedness is essential not only for individual professional development but also for the resilience and effectiveness of Syria's future health workforce.
